# Supplementary material for: Dependence of the shape of graphene nanobubbles on trapped substance
Source: Nat Commun. 2017 Jun 16;8:15844. doi: 10.1038/ncomms15844 (PMC5481778; doi:10.1038/ncomms15844)
Supplement: Supplementary Information [file ncomms15844-s1.pdf]

Type of file: pdf
Size of file: 0 KB
Title of file for HTML: Supplementary Information
Description: Supplementary Notes, Supplementary Figures, Supplementary Table and Supplementary References

Type of file: mpg
Size of file: 0 KB
Title of file for HTML: Supplementary Movie 1
Description: Molecular dynamics simulation of a helium bubble at room temperature. Trapped helium atoms below the bumped graphene move like a gel and the bubble has in general a non-round shape. The graphene substrate is not shown.

Type of file: mpg
Size of file: 0 KB
Title of file for HTML: Supplementary Movie 2
Description: Molecular dynamics simulation of a water bubble at room temperature. The movie shows a top view of the trapped water molecules below the bumped graphene. The graphene substrate is not shown.

Type of file: pdf
Size of file: 0 KB
Title of file for HTML: Peer Review File
Description:

SUPPLEMENTARY NOTE 1. BUBBLE ELASTICITY

Nonlinear equations for an isotropic elastic thin plate is often used to describe the mechanical behavior of monolayer graphene as given by

$$\kappa\nabla^4 z(r) - N_{ij}\frac{\partial^2 z}{\partial x_i \partial x_j} = q\,, \tag{1}$$

where κ is the bending moduli, z(r) is the deformation, $N_{ij} = \frac{Y}{2(1+\nu)}(\varepsilon_{ij} + \frac{\nu}{1-\nu}\delta_{ij})$ is the in-plane membrane force, and q is the lateral loading intensity (usually external pressure). Note that in N_{ij} definition, Y is linear elastic modulus, ν is Poisson's ratio, and ε_{ij} is the strain tensor. Supplementary Equation (1) is independent of the trapped material. When the membrane is subjected to a uniform lateral load, neglecting bending stiffness plus applying clamped boundary condition at the edge (i.e. $z = \frac{dz}{dr} = 0$ at $r = R$), this equation can be solved analytically, giving the vertical displacement

$$z(r) = h_{\max}(1 - (\frac{r}{R})^2) \tag{2}$$

and the radial displacement

$$u(r) = u_0\frac{r}{R}(1 - \frac{r}{R})\,, \tag{3}$$

where $h_{\max}$ is the maximum height, and R is radius of the bubble, u_0 is a parameter determined later, and r is the radial distance. In order to estimate the adhesion energy, we wrote the elastic energy of deformed membrane as a function of the strain tensor components. Using the deformation of graphene described by Supplementary Equations (2,3), and $\varepsilon_{ij} = \frac{1}{2}(\frac{\partial u_i}{\partial x_j} + \frac{\partial u_j}{\partial x_i} + \frac{\partial z}{\partial x_i}\frac{\partial z}{\partial x_j})$, the radial and circumferential strain components are obtained as

$$\varepsilon_r = \frac{u_0}{R}(1 - \frac{2r}{R}) + \frac{2h_{\max}^2 r^2}{R^4}, \quad \varepsilon_\theta = \frac{u_0}{R}(1 - \frac{r}{R})\,. \tag{4}$$

The corresponding in-plane stretching energy density is estimated

$$U_s = \frac{Y}{2(1-\nu^2)}[\varepsilon_r^2 + 2\nu\varepsilon_r\varepsilon_\theta + \varepsilon_\theta^2]\,, \tag{5}$$

$$= \frac{Y}{2(1-\nu^2)}[\frac{4h_{\max}^4 r^4}{R^8} + (\frac{u_0}{R})^2(4 - \frac{12r}{R} + \frac{9r^2}{R^2}) + \frac{4h_{\max}^2 u_0 r^2}{R^5}(1 - \frac{r}{R})]\,. \tag{6}$$

The total energy difference per unit area is due to the strain energy of the membrane and the externally induced the deformation energy by the pressure difference between inside and outside of the bubble. It is determined by

$$dF = dU_s - (p - p_0)dV\,. \tag{7}$$

For a bubble in equilibrium with fixed radius (R), we minimize the energy as follows:

$$\frac{\partial F}{\partial u_0} = \frac{\partial F}{\partial h_{\max}} = 0\,. \tag{8}$$

Using ν=0.16, the applied pressure as a function of maximum deflection and bubble radius is given by Eq. (1) within the main text. Thus the pressure inside the bubble can be determined from measurement of R and $h_{\max}$ assuming P_0 can be ignored. The interface energy ($\pi R^2\Gamma$) is defined by using adhesion energy between graphene and substrate interface, Γ. The equilibrium bubble radius is obtained by balancing the potential energy of the bubble and the adhesion energy of the graphene-substrate interface,

$$\frac{\partial F}{\partial R} = -2\pi R\Gamma\,. \tag{9}$$

This eventually results in the following equations

$$P_{\text{hyd}} \cong 2.85\frac{Yh_{\max}^3}{R^4}, \quad \Gamma \cong 1.79\frac{Yh_{\max}^4}{R^4}\,. \tag{10}$$

Nonlinear plate theory and taking into account the effect of the bending stiffness (κ) that is used in the analysis of graphene bubbles is applied. Using similar approach by treating the graphene monolayer as an elastic plate, the deflection profile and radial displacement can be found to be

$$z(r) = h_{\max}(1 - (\frac{r}{R})^2)^2 \,, \tag{11}$$

$$u(r) = r(R - r)(c - dR) \,, \tag{12}$$

where c and d are parameters that are determined later. For the radial and circumferential strain components we have

$$\varepsilon_r = -Rc + 2(c + Rd)r - 3dr^2 + \frac{8h_{\max}^2 r^2 (R^2 - r^2)^2}{R^8}, \quad \varepsilon_\theta = (R - r)(c - dr) \,. \tag{13}$$

To obtain the strain tensor, we need to consider both in-plane stretching and bending contributions into the energy density, i.e $U = U_s + U_b$ where

$$U_b = \frac{\kappa}{2}[(\frac{d^2 z}{dr^2})^2 + \frac{1}{r^2}(\frac{dz}{dr})^2 + \frac{2\nu}{r}\frac{dz}{dr}\frac{d^2 z}{dr^2}] \tag{14}$$

$$= \frac{16\kappa h_{\max}^2}{R^8}[R^2(1 + \nu)(R^2 - 4r^2) + (5 + 3\nu)r^4] \,. \tag{15}$$

The introduced parameters (c and d) are obtained by minimizing the total energy $\frac{\partial F}{\partial c} = \frac{\partial F}{\partial d} = 0$. Consequently, $\frac{\partial F}{\partial h_{\max}} = 0$ and assuming $\nu = 0.16$ gives us the pressure as

$$P_{\text{hyd}} \cong 2.56\frac{Y h_{\max}^3}{R^4} + 64\frac{\kappa h_{\max}}{R^4} \,. \tag{16}$$

Finally, using Supplementary Equation (9), we obtained the adhesion energy which is given by

$$\Gamma \cong \frac{Y h_{\max}^4}{R^4} + 32\frac{\kappa h_{\max}^2}{R^4} \,. \tag{17}$$

Moreover, the stress-strain components σ_r and σ_θ for a 2D plate are given by

$$\sigma_r = \frac{Y}{(\nu + 1)(2\nu - 1)}[(\nu - 1)\varepsilon_r - \nu\varepsilon_\theta] \,, \tag{18}$$

$$\sigma_\theta = \frac{Y}{(\nu + 1)(2\nu - 1)}[(\nu - 1)\varepsilon_\theta - \nu\varepsilon_r] \,. \tag{19}$$

Substituting ε_r and ε_θ from Supplementary Equation (13) into Supplementary Equations (18,19), each separately results in a polynomial function of $x = r/R$ defined by

$$f_\sigma(x) = 1 + Ax + Bx^2 + Cx^4 \tag{20}$$

where A, B, and C are coefficients depending on the elastic constants. Supplementary Table 1 shows the corresponding fitting coefficients of the ethanol bubble discussed within main text (Stress Calculations).

Supplementary Table 1: Fitting parameters of $f_\sigma(x)$ for ethanol bubble (x <1) based on MD data (σ_{rr} and $\sigma_{\theta\theta}$) and quality factor Q.

Fit Coeffs.	$f_{\sigma_{rr}}(x)$	$f_{\sigma_{\theta\theta}}(x)$
A	0.0759	0.265
B	-1.068	-3.098
C	0.396	1.547
Q	0.994	0.987

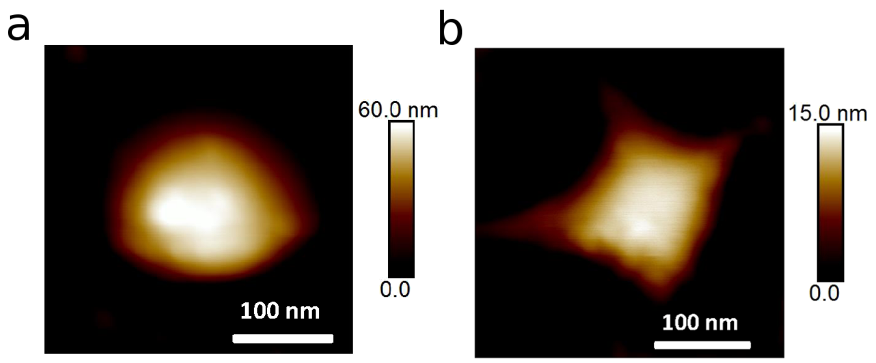

Supplementary Figure 1: **AFM images of the water bubble.** (a-b) two typical AFM images of a water nanobubble.

SUPPLEMENTARY NOTE 2. BUBBLE VOLUME AND RADIUS CALCULATION

The pressure (P) and density (ρ) of the materials inside the bubble crucially depend on how accurately we determine the bump volume V_b using Eqs. (4,5) within the main text. Furthermore, measuring the height and radius of the bubble is important to obtain the morphology of the bubble. To calculate the volume of the bubble, the top graphene layer is fitted by a function $h(x, y)$ determine the time average xyz coordinate of the carbon atoms. Because not the whole space of the volume is accessible to the trapped atoms -due to repulsive potential between graphene and the atoms- we defined the effective height function $h_\mathrm{eff}(x, y)$ which excludes the volume related to the distance between the molecule-graphene ($\sigma_\text{mol-C}$):

$$h_\mathrm{eff}(x, y) = h(x, y) - \sigma_\text{mol-C} \,, \tag{21}$$

where $\sigma_\text{mol-C}$ is the average LJ parameter between trapped molecules and C atoms. Then, we estimated the volume and the radius by integrating over the bump area using the following equations:

$$V = \int_\mathrm{bump} h_\mathrm{eff}(x, y) dx dy \,, \tag{22}$$

$$A = \int_\mathrm{bump} dx dy \;\Rightarrow\; r = \sqrt{A/\pi} \,. \tag{23}$$

The bump area is defined by the estimated region where the trapped atoms exist. We admit that without corrections for the effective volume of the bump due to difficulty in estimating the exact accessible volume for the trapped atoms, MD overestimates the measured volume. Consequently, it underestimates the pressure and the density. This effect becomes important for small bubbles when the size of the excluded volume is comparable to the total volume.

SUPPLEMENTARY NOTE 3. ATOMIC FORCE MICROSCOPY MEASUREMENTS

We depict the AFM images of two typical water bubbles and four typical hydrocarbon bubbles which the (none-) round-shape hydrocarbon bubbles can be seen from the images shown in Supplementary Figures 1(a-b) and 2(a-d).

SUPPLEMENTARY NOTE 4. SOLVENTS

We have used water, ethanol and methanol as solvents to fabricate solvent filled graphene nanobubbles. We presented the data and AFM images of water/ethanol filled with graphene nanobubbles in Figs. 4(a,b) and 7(a,b) within the main text. We have also carried out Raman spectroscopy as a characterization technique to probe the presence of trapped substances. As a reference, here we have shown the Raman spectrum of bulk methanol/ethanol and methanol/ethanol filled graphene nanobubbles (see Supplementary Figures 3(a,b)). It is clear that all the Raman bands related to the methanol/ethanol (indicated as red color arrow marks in the inset figures) are present in the corresponding nanobubbles with small shifts in the wavenumbers.

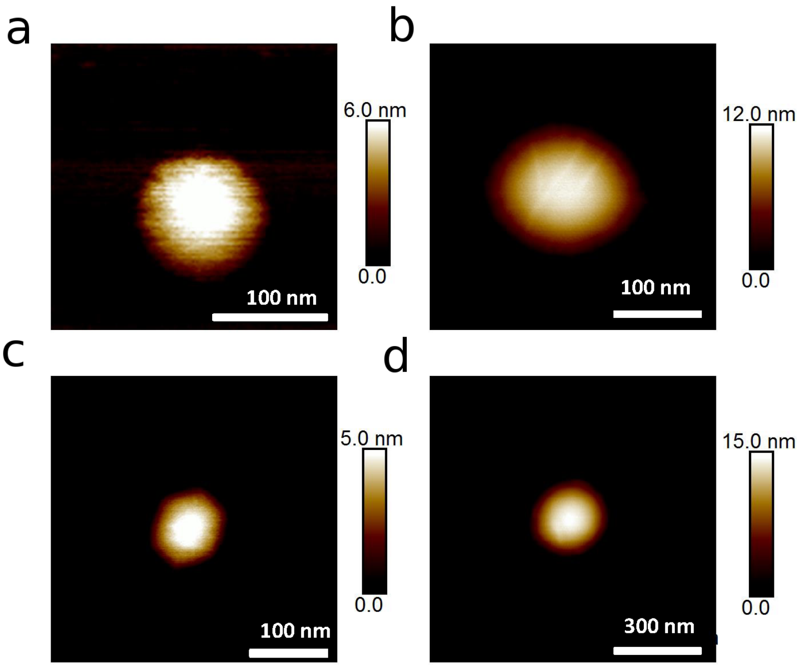

Supplementary Figure 2: **AFM images of the ethanol bubble.** (a-d) four typical AFM images of hydrocarbon nanobubble.

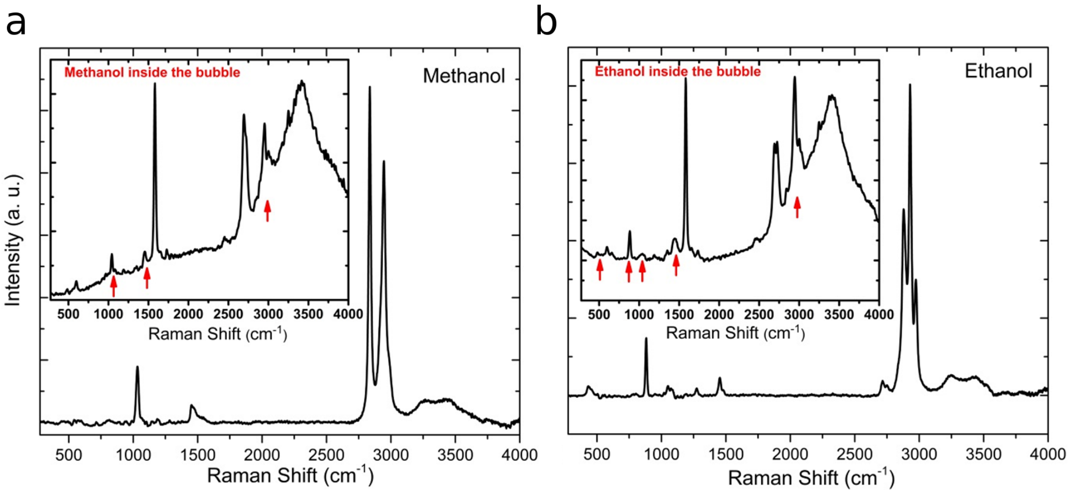

Supplementary Figure 3: **Raman spectrum of hydrocarbons.** (a) and (b) respectively shows Raman spectrum of bulk methanol/ethanol and methanol/ethanol filled graphene nanobubbles (Inset figures).

SUPPLEMENTARY NOTE 5. DEFORMATION PROFILE

For the deformation profiles of the graphene sheet, based on elastic theory, it is found that it can be fitted by a polynomial function:

$$f(x) = 1 - x^2 + \alpha(x^2 - x^4), \quad x \in [-1, 1], \tag{24}$$

where $x = r/R$ and r is the radial distance from the center of the bubble. Notice that $f(\pm 1) = 0$, and the $|\alpha|$ parameter determines the strength of the nonlinear elasticity of the top graphene sheet and it is taken as a fitting parameter (Ref. [1]). In Supplementary Figure 4, we show the height-profile from our MD simulations and the fit function $f(x)$ for helium (a), water (b), and ethanol (c) at room temperature. In Supplementary Table 2, we provide

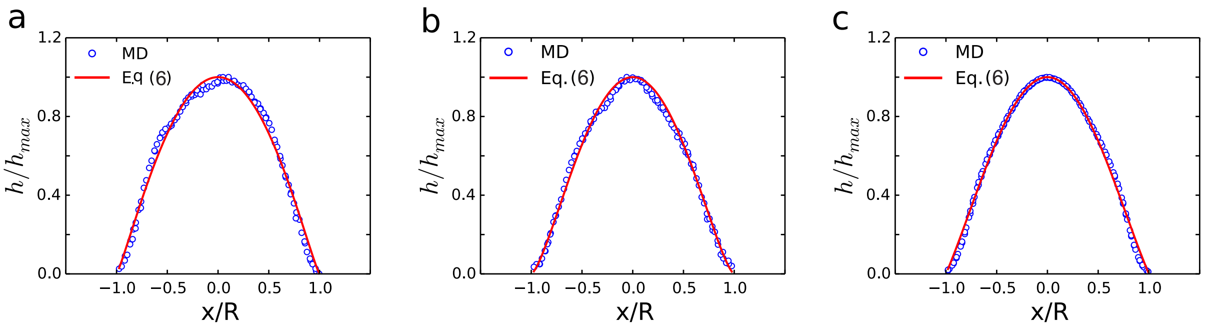

Supplementary Figure 4: **Bubbles deformation profile.** Three horizontal cross sections from graphene bumps containing (a) helium, (b) water and (c) ethanol at 300 K with corresponding fitting function given in Supplementary Equation (24).

a list of the corresponding fit parameters for the studied polar liquids and helium at 0 K and 300 K. The sign of α is consistent with the prediction of $\alpha = -1$ (Ref. [1]), however its value is different. The smaller $|\alpha|$ value for ethanol indicates smaller nonlinear effects (and bending stiffness effects). Notice that the deformation profiles for hydrocarbon bubbles have higher quality factor – Q – which is defined by $Q = 1 - \sqrt{\frac{1}{N}\sum_{i=1}^{N}(h_i/h_{\max} - f(x_i))^2)}$ leading us to conclude that the experimental bubbles are filled by hydrocarbons rather than water and helium.

Supplementary Table 2: Fitting parameter α presented in Supplementary Equation (24) at zero and room temperature for polar liquids and helium, where Q is the quality factor of the fit and Q=1 corresponds to perfect fit.

Trapped substance	0 K		300 K	
	α	Q	α	Q
Helium	-0.56	0.967	-0.21	0.966
Water	-0.37	0.972	-0.54	0.978
Ethanol	-0.13	0.981	-0.39	0.984

SUPPLEMENTARY NOTE 6. THE EFFECT OF BUBBLE SIZE ON DENSITY PROFILE

Supplementary Figure 5(a) shows the average density profile of oxygen atoms along the z-axis for two typical ethanol bubbles with sizes of 150 (blue squares) and 200 (red circles) molecules at 0K. The peak(s) indicate the layered structure of trapped atoms. By increasing the number of atoms more layers are formed. Similarly, density of oxygen atom along z-axis for two typical water bubbles with sizes of 800 (blue squares) and 2300 (red circles) molecules at 0K are shown in Supplementary Figure 5(b).

SUPPLEMENTARY NOTE 7. THE EFFECTS OF BOUNDARY STRESS

Finally, we studied a strained bubble and its effect on the trapped water structure. In order to induce boundary stress, we carried out two additional simulations: i) fixed the top graphene sheet atoms beyond a circle with radius 15 nm, and ii) we similarly fixed the top graphene sheet atoms beyond a square block of size $15\times15\,\text{nm}^2$. The former is equivalent to inducing a triaxial stress Ref. [3] ($\sigma_{xx} = -\sigma_{yy}$, $\sigma_{xy} = \sigma_{yx}$) and the later corresponds to the application of biaxial stress ($\sigma_{xx} = \sigma_{yy}$, $\sigma_{xy} = \sigma_{yx} = 0$). The bubble, subjected to such boundary stress, forms trigon and linear wrinkles and the optimized structure are shown respectively in Supplementary Figures 6(a,b). Wrinkles and ripples are commonly observed in experiment. Such boundary stress fundamentally changes the shape of the bubble (Refs. [4–6]). The circular bubbles filled with water are found to be elongated and flattened due to the applied boundary stress.

SUPPLEMENTARY NOTE 8. THE EFFECT OF ADHESION ENERGY

To investigate the effect of the van der Waals pressure on the structure of the trapped atoms, we performed several simulations using an extra Lennard-Jones (LJ) interaction between the graphene substrate and the cover layer in case

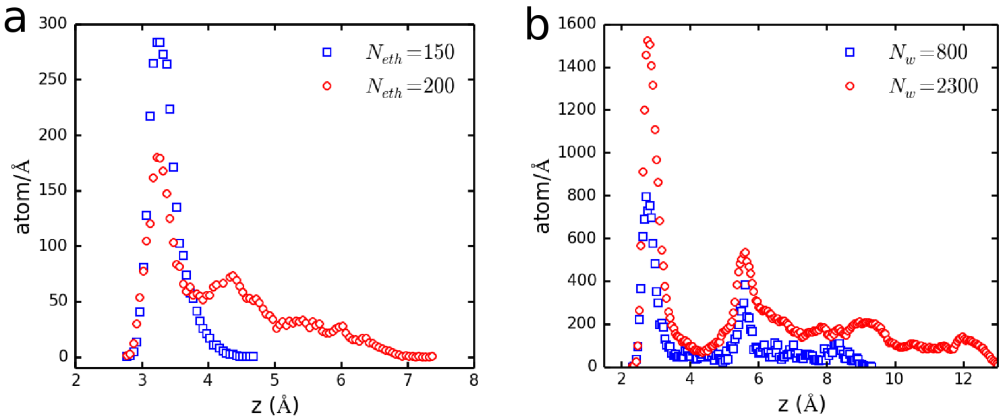

Supplementary Figure 5: **The effect of bubble size on the density profile of the trapped materials.** The average density profile of oxygen atoms along z-axis for two different number of trapped molecules for (a) ethanol and (b) water nanobubble.

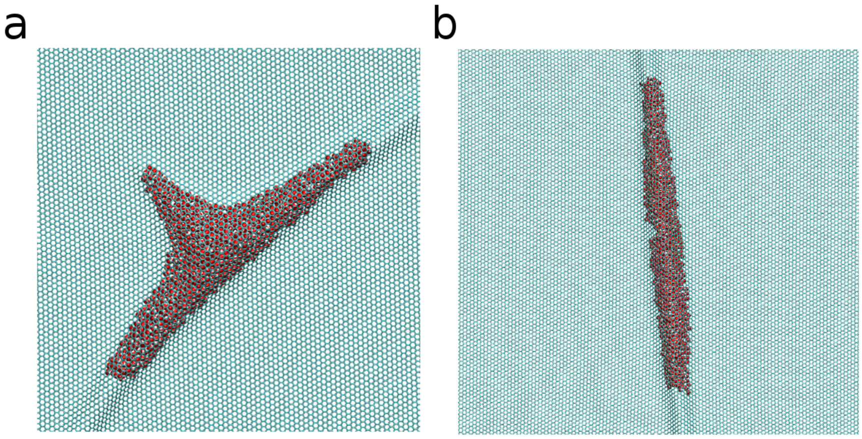

Supplementary Figure 6: **The effects of boundary stress.** Top view of trigon (a) and linear (b) wrinkle corresponding to triaxial and biaxial stress at T=0. The water molecules (dark particles) are partially distributed within the wrinkles. Top graphene sheet is fixed beyond the circular region in (a) and a square region in (b) resulting in different types of wrinkle formation.

of a water bubble with 792 water molecules at room temperature. In fact by tuning the energy parameter (ϵ) in the LJ potential, we have been able to elucidate the effects of adhesion between the two graphene layers (outside the nanobubble region). Note that increasing the adhesion energy is equivalent to a large vdW pressure.

We calculated the RDF of trapped water for several values of ϵ at room temperature. The second and third peaks become more pronounced when the depth of the potential well in the LJ potential deepens from 0 to 100 meV (see Supplementary Figure 7). This is a signature of increasing long-range ordering due to the crystalline structure of nanoconfined water. Even by increasing the adhesion energy between the two graphene sheets, which is equivalent to increasing the vdW pressure, a more ordered structure for water is obtained, however the square ice structure and its stacking structure are not seen. This casts some doubts on the observation of square ice in Ref. [2]. In contrast, based on our MD data for salt bubble and corresponding RDF with first peak at 2.8 Å, we argue that the observed square structure belongs to face-centered cubic NaCl lattice.

SUPPLEMENTARY REFERENCES

[1] Yue, K.; Gao, W.; Huang, R.; Liechti, K. M. Analytical methods for the mechanics of graphene bubbles. *J. Appl. Phys.* **112**, 083512-083519 (2012).
[2] Algara-Siller, G. *et al.* Square ice in graphene nanocapillaries. *Nature* **519**, 443-445 (2015).

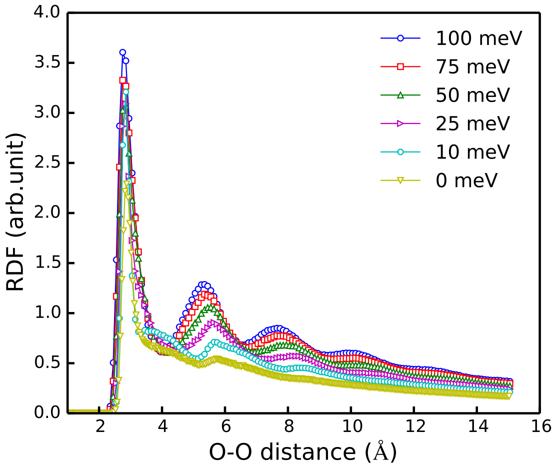

Supplementary Figure 7: **The effect of adhesion energy.** The O-O radial distribution of confined water molecules for different LJ interaction strength (ϵ) between two graphene layers at room temperature.

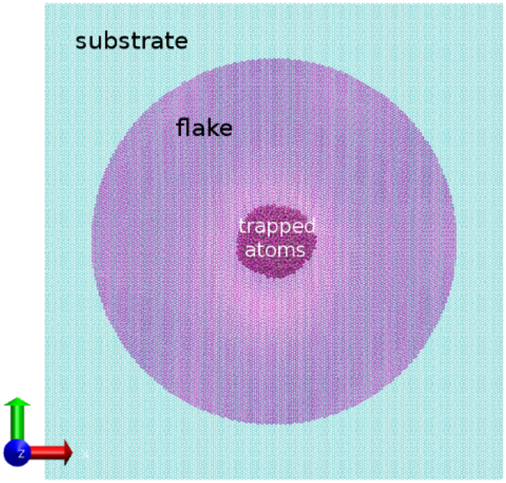

Supplementary Figure 8: **MD model.** Top view of setup model for our MD simulation.

[3] Neek-Amal, M.; Covaci, L.; Shakouri, K.; Peeters, F. Electronic structure of a hexagonal graphene flake subjected to triaxial Stress. *Phys. Rev. B* **88**, 115428-115436 (2013).

[4] Calado, V.; Schneider, G.; Theulings, A.; Dekker, C.; Vandersypen, L. Formation and control of wrinkles in graphene by the wedging transfer method. *Appl. Phys. Lett.* **101**, 103116-103118 (2012).

[5] Wang, C.; Liu, Y.; Lan, L.; Tan, H. Graphene wrinkling: formation, evolution and collapse. *Nanoscale* **5**, 4454-4461 (2013).

[6] Deng, S.; Berry, V. Wrinkled, rippled and crumpled graphene: an overview of formation mechanism, electronic properties, and applications. *Mater. Today* **19**, 197-212 (2016).
